# P-1610. Assessing the Impact of Reflex Urine Cultures in Pediatrics

**DOI:** 10.1093/ofid/ofae631.1777

**Published:** 2025-01-29

**Authors:** Lisa Saiman, Karen P Acker, Harjot K Singh, Lars Westblade, Hannah I Joyce, Xiaoyue Ma, David Kuang, Adam L Gouveia, Barbara Ross, Shirley Wang, WooJin Shin, Nicole Gerber, Michael J Alfonzo, Samiksha Tarun, Taylor Dempsey

**Affiliations:** Columbia University Irving Medical Center, New York, NY; Weill Cornell Medicine/New-York Presbyterian, New York, NY; Weill Cornell Medicine, new york city, New York; Weill Cornell Medicine, new york city, New York; Columbia University Irving Medical Center, New York, NY; Weill Cornell Medicine, new york city, New York; NEW YORK PRESBYTERIAN, HOBOKEN, New Jersey; NewYork-Presbyterian, Brooklyn, NY; NewYork-Presbyterian Hospital, New York, NY; NYP, new york, New York; Columbia University Irving Medical Center, New York, NY; NYP - Weill Cornell, New York, New York; Weill Cornell Medicine, new york city, New York; Columbia, New York, New York; New York Presbyterian/Weill Cornell, New York, New York

## Abstract

**Background:**

Obtaining a urine culture (UCx) in the absence of urinary symptoms and inflammation has been shown to result in misdiagnosis and over treatment of urinary tract infections (UTIs) and catheter-associated UTIs in adults. Reflex UCx is a diagnostic stewardship strategy to reduce unnecessary UCx by performing UCx only if a urinalysis (UA) meets criteria for infection. Although the AAP has recommendations for reflex UCx in pediatrics, there are little data to support this approach. We aimed to assess the potential impact of reflex UCx on reducing unnecessary UCx in children.

Age, specimen type, and hospital location in children with positive and negative UCx.

**Figure:**
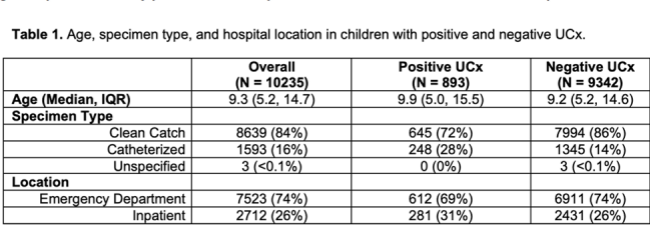

**Methods:**

We performed a retrospective review of children age 2 to 18 years at our network of 8 hospitals from the emergency department or inpatient settings from 11/1/2020 to 7/20/2023. We included children with a matched microscopic UA (mUA) and UCx on the same calendar day. Positive UCx was defined as ≥ 50,000 CFU/ml for catheterized specimens and ≥ 100,000 CFU/ml for clean catch or unspecified specimens. Pyuria was defined as ≥ 10 WBC/HPF. Manual chart review was performed for positive UCx when < 10 WBC/HPF to determine cases consistent with clinical UTI. Sensitivity, specificity, positive predictive value, and negative predictive value with 95% confidence intervals were calculated.

Sensitivity, Specificity, Positive Predictive Value (PPV) and Negative Predictive Value (NPV) of Pyuria Compared to Positive Urine Culture and Clinical UTI.

**Figure:**
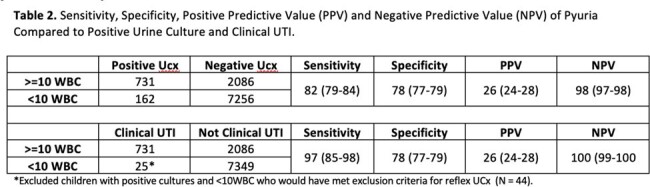

**Results:**

During the baseline period, 10,235 patients had a mUA and UCx, of which 893 (8.7%) UCx were positive. Median age, specimen types and hospital location are listed in Table 1. Of the positive UCx, 162 (18%) were associated with < 10 WBC/HPF. Of these cases, 44 (27%) had conditions that would have excluded them from reflex UCx per our hospital guideline: 36 (22%) had urologic abnormalities, 5 (3.1%) were pregnant, 3 (1.9%) were renal transplant recipients, and 0 (0%) had neutropenia. Of the remaining 118 positive cultures with < 10 WBC/HPF, 93 (79%) were not consistent with clinical UTI. Table 2 shows the sensitivity and negative predictive value (NPV) of pyuria for a positive UCx and clinical UTI.

**Conclusion:**

In our study, we demonstrated the safety of a reflex UCx approach in a large pediatric population. Reflex UCx had low risk for missing clinical UTIs and would have reduced the number of unnecessary UCx during the pre-implementation period as 7349 (72%) would not have been performed. Reflex UCx should become standard of care in pediatrics.

**Disclosures:**

**Lars Westblade, PhD**, Accelerate Diagnostics, Inc: Grant/Research Support|bioMerieux, Inc: Grant/Research Support|Element Materials Technology: Grant/Research Support|Hardy Diagnostics: Grant/Research Support|Roche Molecular Systems, Inc.: Advisor/Consultant|Roche Molecular Systems, Inc.: Grant/Research Support|Selux Diagnostics, Inc.: Grant/Research Support|Shionogi, Inc: Advisor/Consultant|Talis Biomedical: Advisor/Consultant

